# The International Congress at Buda Pesth

**Published:** 1894-09-22

**Authors:** 


AN INSTITUTIONAL JOURNAL OF

The Medical Sciences and Hospital Administration,

WITH

VOL. XVI.—No. 417.] AN EXTRA NURSING SUPPLEMENT. [SEPT. 22, 1894.

### THE INTERNATIONAL CONGRESS AT BUDA PESTH.

NOTWITHSTANDING that the American Dr. Billings protested against the turning of this International Congress at its close into a scientific Tower of Babel, notwithstanding that the correspondent of the English *Times* foresees the extinguishment of such congresses by the neglect of the leaders of science unless sweeping amendments of procedure are made, it is certain that some of the most profoundly interesting scientific problems of the period were competently discussed at Buda Pesth, and that many of the discussions will bear practical fruit in laboratories, at the bedside, and over wide areas of social activity for many years to come. No doubt too much was attempted. Too much always is attempted on such occasions. But even managers of scientific congresses are mortal, and when the ambition to read papers is rampant, and large temptations to pleasure are thrust upon the indispensable listener, it is easy to imagine a result disappointing to the serious worker, whose capacity for movement and variety of occupation is limited by age or other physiological necessity. Judged, however, by the character as well as by the extent of the work done, it cannot be denied that the Congress will bear comparison with most of its predecessors for the interest and enthusiasm it undoubtedly evoked, and for the permanent stimulus it has given to scientific activity.

The Congress met for a week, and accepted more than 800 papers for discussion in 26 sections. It was quite impossible that this enormous number of papers could be so much as read, much less fully discussed. But of the papers that were read and discussed several were of such deep, immediate, and lasting interest that their consideration amply redeemed the whole Congress from the charge of failure. The practising physician and the hygienic philosopher alike turn with the keenest interest to the papers on diphtheria and cholera. The reports on diphtheria presented to the Congress by Dr. Edward Seaton on behalf of the English, Dr. Billings on behalf of the American, and Professor Loeffler on behalf of the German Committee, bring us face to face with a set of facts which show, with the distinctness of scientific certainty, both the extent of our knowledge and the depth of our ignorance, both the limitations of our power and the wide reach of our control over some of the most insidious and deadly enemies of mankind. Dr. Billings and Dr. Edward Seaton made it plain beyond dispute that in America and England the ravages of diphtheria have been extending during the past twenty years. This increase has been more pronounced in England than in America, and especially in large towns. A glance over the tabulated statistics shows that the increase in England has been almost unintermittent, and that in not a few urban districts the number of diphtheria cases has more than doubled within the twenty years, and that the fatality has correspondingly increased.

What has civilisation, what has science to say about facts of this kind, which when looked intelligently in the face may properly be described as "tremendous"? There has never been known in the world's history a twenty years period of wider and more strenuous scientific activity than the twenty years period just passed; and yet it is precisely during these twenty years of the most strenuous and most intelligent physiological and scientific activity that the incidence of diphtheria and its fatality have in many instances almost or quite doubled. It is impossible for the physician, who comprehends and takes time to consider, to be jubilant over facts like these. They are huge facts, facts which extend over countries and continents, facts which concern the vitality and the very existence of the new generations of men; they are facts which the physician who takes note of the people as well as of himself and his own patients regards not only with stern dissatisfaction, but also with perplexed alarm. It is true that Dr. Edward Seaton thinks he has discovered the chief source of this new danger which threatens civilisation in the too indiscriminate massing together of children in elementary schools; it is true that Professor Loeffler presented with his report an elaborate series of proposed checks and preventive measures for the limitation of so deadly a disease as diphtheria within the narrowest possible bounds. But the practical physician sees that whilst it is not quite certain that the real *fons et origo mali* has been discovered, and whilst the various methods of prophylaxis propounded by Professor Lœffler belong to a large extent to the class of the "great unproved"; diphtheria is still spreading on all hands and increasing in its fatality. The problem is of the gravest, and the Congress did well, exceedingly well, in that it presented the full and striking facts for the consideration of civilisation and scientific medicine.

Dr. Billings made an attempt to solve the ancient problem of the identity or non-identity of diphtheria and croup. The pendulum, which not many years ago swung towards non-identity, and still more recently swung with great decision towards identity, is now swinging back again with still more authority towards non-identity. The question whether or not the bacillus diphtheriæ is present in any given membranous sore-throat is decided to be the new diagnostic determinator. But, indeed, does not the whole discussion show that an entirely new nomenclature and an entirely different and more philosophical attitude of mind are the real and imperative necessities of the hour and of truly progressive science? What progress can ever be made so long as we are hampered and hindered by such a muddle of terms and definitions that no scientist is ever quite sure what any other scientist means? We must give up much of the medical lumber of the past; we must begin, again and again, and again, from the foundation, if definite ground is to be gained, and still more if it is to be retained.

The antitoxin treatment of diphtheria, of which M. Roux gave an enthusiastic account, is novel, so far as anything of this kind can now be considered novel, and the results said to have been gained were very striking. But we confess to a settled determination to receive every announcement of new and startling cures, from whatever source it may come, with unmoved caution. We shall try the antitoxin treatment in England, as we tried tuberculin; and we shall try it fairly. When we shall have made a sufficient induction of particulars we shall be prepared to give a verdict, and it is pretty certain that the verdict of England will be final. If the therapeutic wings of the British physician are not quite so swift as those of some of our continental brethren, it is quite certain that they are much more sure.

The cholera discussion which was introduced by Dr. Elias Metchinkoff proved a thorny but exceedingly interesting subject. The ink which announced the doctrine of phagocytosis is hardly yet dry on the page, and the voices of the eminent scientists who applauded such an advance in physiology are still ringing in our ears; but all this notwithstanding, Dr. Buchner and others insisted that "phagocytosis is a secondary phenomenon," and that immunity, which may be natural or artificial, or both, the former being due to alexins and the latter to antitoxins, is only in a strictly limited sense the result of the action of phagocytes. Here, again, we have a return to earlier beliefs, though these beliefs are now held to have been rationalised by recent physiological research and discovery. The Congress, in short, was intensely alive at its centre, however much it may have got out of hand at its circumference. The moral both of its success and of its failure is that in future Congresses the business arrangements must be entrusted to business men, and that whilst the picnic element is not to be entirely tabooed, it must nevertheless constitute the recreation of the assembly, whilst serious and sustained work must be its distinguishing characteristic.

